# Pulp-Derived Exosomes in a Fibrin-Based Regenerative Root Filling Material

**DOI:** 10.3390/jcm9020491

**Published:** 2020-02-11

**Authors:** Anja Ivica, Chafik Ghayor, Matthias Zehnder, Silvio Valdec, Franz E. Weber

**Affiliations:** 1 Oral Biotechnology and Bioengineering, Center for Dental Medicine, University of Zurich, 8032 Zurich, Switzerland; anja.ivica@zzm.uzh.ch (A.I.); chafik.ghayor@usz.ch (C.G.); 2 Preventive Dentistry, Periodontology and Cariology, Center for Dental Medicine, University of Zurich, 8032 Zurich, Switzerland; matthias.zehnder@zzm.uzh.ch; 3 Cranio-Maxillofacial and Oral Surgery, Center for Dental Medicine, University of Zurich, 8032 Zurich, Switzerland; silvio.valdec@zzm.uzh.ch

**Keywords:** exosomes, dental pulp, regenerative endodontics, fibrin gel

## Abstract

Regenerative endodontics has been described as a paradigm shift in dentistry, despite its current limitation to immature teeth and reparative rather than regenerative outcomes. Cell-free treatments are favored because of regulatory issues. However, the recruitment of host-derived stem cells to the desired site remains challenging. We investigated whether dental pulp-derived exosomes, which are extracellular vesicles that contain proteins, lipids, RNA, and DNA and thus mirror their parental cells, may be used for this purpose. The use of exosomes may present appreciable advantages over the direct use of transplanted stem cells due to a higher safety profile, easier isolation, preservation, and handling. Here we harvested exosomes from a cultured third-molar pulp cell and assessed them by transmission electron microscopy and Western blotting. Human mesenchymal stem cells (MSCs) were exposed to these exosomes to assess exosome uptake, cell migration, and proliferation. In addition, a fibrin gel (i.e., a diluted fibrin sealant), was assessed as a delivery system for the exosomes. Our results show that exosomes attracted MSCs, and the fibrin gel enhanced their effect. Moreover, exosomes improved the proliferation of MSCs. Therefore, we propose that pulp-derived exosomes in combination with a fibrin gel could be a powerful combination for clinical translation towards improved cell-free regenerative endodontics and thus represent a new way to fill dental hard tissues.

## 1. Introduction

In theory, regenerative treatments in endodontics should offer many potential advantages over traditional clinical concepts, which merely arrest the progress of the disease and rely on prosthetic materials to seal off remaining dental hard tissues against the oral cavity. However, the current “regenerative” treatment approach is limited to revascularization of necrotic pulp spaces in immature roots of young teeth with open apices. It suffers from multiple shortcomings such as blood-derived tooth discoloration and the inability to regenerate a functional dental pulp [1]. Consequently, regenerative endodontics may still be an overly optimistic term, as treatment outcomes are reparative rather than regenerative. Nevertheless, the American Association of Endodontists and the European Society of Endodontology recognize these treatments as valid [2,3]. The approved protocol relies on cell homing and includes disinfection of the pulp space using a sodium hypochlorite solution followed by dentin conditioning using a decalcifying agent such as EDTA to release growth factors from the hard tissue phase of dentin [2,3,4]. One of the main challenges in this strategy is to recruit the patient’s endogenous mesenchymal stem cells (MSCs) into the disinfected and conditioned pulp space [5]. This may explain why clinical results are rather unpredictable [6].

Alternatively, MSCs are proposed as a therapy in many clinical indications [7]. When they reach the site of injury, MSCs release cytokines and growth factors that stimulate host progenitors to divide and differentiate into more specialized cells to produce new functional tissue [8]. The transplantation of dental pulp cells or other stem cells into an empty canal induces the formation of pulp-like tissue [9,10,11]. However, the costs of the lab work, cell expansion, and delivery make this treatment unattractive to patients. In addition, there are risks of contamination, tumorigenesis, and immunorejection [12]. 

On the other hand, the cell homing approach, which relies on the recruitment of host-derived stem cells from the apical part of the root, periodontium, or blood, is a cell-free approach and a good alternative to cell transplantation strategies [4]. A major disadvantage, however, may be the possible shortage of recruitable cells [12]. Recent research attempted to improve stem cell recruitment into the pulp space and subsequent cell differentiation by implementing chemokine delivery systems in regenerative endodontic procedures [4,13,14]. Another option could be to use dental pulp-derived exosomes in regenerative procedures [15]. Recently, there was a paradigm shift concerning the use of MSCs, after it was shown that exosomes harvested from MSCs show a plethora of biological activities, which are rather similar to activities achieved with MSCs alone [16]. Exosomes represent a key concept in mediating changes in cellular behavior through genetic material transfer since they contain miRNA [17]. Depending on their origin, exosomes act and operate differently [18]. Hitherto, 129 clinical trials using exosomes are listed in www.clinicaltrials.gov. A further advantage of using exosomes instead of mesenchymal stem cells is that they represent a cell-free therapy, and thus avoid immune responses and other possible untoward effects [19,20]. The use of exosomes may present appreciable advantages over the direct use of MSCs due to a higher safety profile and lower immunogenicity [21]. The ability of dental pulp exosomes to trigger lineage specific differentiation has been shown [15]. However, the involvement of exosomes to help in stem cell recruitment and the choice of the delivery system for exosomes have not been studied yet. 

To implement morphogenic molecules into endodontic procedures, a matrix material is required to replace the patient-derived blood clot. Most similar to a blood clot is a fibrin hydrogel, which can be obtained by diluting a commercially available fibrin sealant in order to get an ideal space between fibers for cell migration [22,23]. A fibrin gel showed superior properties over different natural and synthetic scaffolds to form pulp-like tissue [24]. Moreover, fibrin is a well-known delivery system for the release of tissue factors also in conjugation with pulp regeneration [13,25]. The combination of a fibrin sealant and cells is used for many tissue-engineering approaches [26]. In the context of exosomes, the application of a mixture of exosomes and fibrin glue was successfully demonstrated for the treatment of incisional hernia [27].

Based on the background information delineated above, the aims of this study were to assess if exosomes from dental pulp cells trigger the migration of stem cells. Furthermore, we studied the interactions between fibrin gel as a potential delivery system for exosomes and the activity of exosomes in terms of cell migration.

## 2. Materials and Methods 

### 2.1. Human Pulp Cells

Healthy third molars (*n* = 3, 16 to 25 years old) were extracted for reasons not related to this study at the Center of Dental Medicine, University of Zurich. All respective patients had impacted teeth and gave informed consent that their teeth would be used for scientific purposes. Anonymized tooth samples obtained with written informed consent are exempt from the necessity of obtaining an individual ethics approval according to local law (Swiss Federal Council, Federal Act on Research involving Human Beings). Extractions were done carefully in order to minimize the risk of infection. Freshly extracted teeth were kept in Dulbecco’s modified Eagle’s medium (DMEM, Gibco, Paisley, UK) supplemented with 10% Pen Strep, and 10% fetal bovine serum (FBS, Gibco, Paisley, UK).

The tooth crown was removed using a diamond bur under water-cooling. The pulp tissue contained in the tooth was then harvested and cut into smaller pieces, washed in phosphate-buffered saline (PBS, Gibco), and finally digested in a collagenase/dispase solution (Roche, Mannheim, Germany). Cells were cultured in DMEM supplemented with 1% Pen Strep and 10% fetal bovine serum (FBS, Gibco). Cells at passages 3–6 at 80% confluence were used in the following experiments.

### 2.2. Exosome Isolation

When the dental pulp cells (DPCs) became confluent, the cell medium was exchanged by an exosome-free medium (Invitrogen, Carlsbad, CA, USA). After 48 h, cell culture supernatants were harvested, centrifuged at 300× *g* for 10 min and 2000× *g* for 30 min to remove cells and debris, and a total exosome isolation agent (Invitrogen) was added. The following day the mixture was centrifuged at 10,000× *g* for 30 min and the pellet was resuspended in PBS. 

### 2.3. Negative Staining for Transmission Electron Microscopy (TEM)

To verify the presence and the size of exosomes, TEM analysis was performed. Freshly isolated exosomes were used for this experiment. A drop of sample (10 µL) was pipetted on top of a copper grid (G2300C, Plano GmbH, Wetzlar, Germany). After washing with PBS, the sample was incubated in 1% glyceraldehyde for 5 min, washed again, and incubated in 1% uranyl acetate for 5 min. The grid was then transferred to methylcellulose stain on ice for 10 min. The methylcellulose contrasting stain was prepared from 100 µL 3% uranyl acetate and 900 µL 2% methylcellulose. An eyelet was then used to lift the grid from the drop of stain. Excess staining solution was removed using a filter paper and air-dried over ice. The sample was observed using a TEM CM100 microscope (Thermo Fisher, Waltham, MA, USA) at accelerating voltage of 80 kV. ImageJ (National Institutes of Health, Bethesda, MD, USA) was used to calculate the diameter of isolated particles. 

### 2.4. Western Blot 

Total cell lysates of the DPCs and exosomal proteins were extracted using RIPA ((Radioimmunoprecipitation Assay) buffer supplemented with protease and phosphatase inhibitors. The samples were loaded on a 4%–20% precast polyacrylamide gel to start the electrophoresis and the gels were transferred to a PVDF membrane using the precast Trans-Blot turbo stack (Bio Rad, Hercules, CA, USA). Blocked with 5% non-fat milk in PBS containing 0.1% Tween-20, the membrane was cut and incubated with either the primary antibodies against CD9 or Grp94 (1:1000; Cell Signaling Technology, Danvers, MA, USA). An anti-rabbit IgG HRP (horse radish peroxidase)--linked antibody (1:2000; Cell Signaling Technology) was used as the secondary antibody. The signals of the proteins were detected using Clarity Western ECL (Enhanced Chemiluminescence) substrate in conjunction with the ChemiDoc MP imaging system (Bio Rad).

### 2.5. Human Bone Marrow-Derived Mesenchymal Stem Cells (HBMMSCs)

The stem cells were purchased from American Type Culture Collection (PCS-500-012; Manassas, VA, USA). They were cultured in DMEM supplemented with 1% Pen Strep and 10% FBS (Gibco). Cells at passages 3–5 were used. 

### 2.6. Uptake of Exosomes by HBMMSCs

BODIPY® TR ceramide (Thermo Fisher Scientific, Waltham, MA, USA) diluted in DMSO (1 mM solution) was used for in vivo labeling of exosomes. DPCs were cultured in standard conditions and the dye solution was added to the flask (1 µL/mL). After 20 min of incubation, the medium was removed, the cells were washed with PBS three times and fresh medium containing exosome-depleted FBS (Invitrogen) was added. The cells were cultured for another 48 h. Afterwards labeled exosomes were isolated from the cell medium using the total exosome isolation agent (Invitrogen) as previously described. 

HBMMSCs were cultured in an 8-well Chamber Slide System (Ibidi, Planegg, Germany). The fluorescently-labeled exosomes were added to HBMMSCs. After 3 h of incubation, the cells were fixed with 4% paraformaldehyde at room temperature for 20 min, permeabilized with 0.1% Triton X-100 (Sigma-Aldrich, St Louis, MO, USA), and stained with Alexa Fluor 488 phalloidin (Thermo Fisher Scientific). After washing in PBS, the chambered coverslip was removed, and the samples were mounted in ProLong Gold Antifade Reagent with DAPI (Cell Signaling Technology). The samples were analyzed with a fluorescence microscope (BX60; Olympus, Tokyo, Japan). For the negative control, only cells without labeled exosomes were used.

### 2.7. Fibrin Gel Preparation 

Fibrin gel was prepared from a commercially available fibrin sealant (Tisseel, Baxter; Deerfield, IL, USA) that consists of fibrinogen-based and thrombin-based solutions. The thrombin component was diluted in Tris-buffered saline pH 7.5 (TBS) at a 1:125 (*v*/*v*) ratio, while the fibrinogen component was diluted 1:5. The diluted solutions were mixed 1:1 to form a gel. 

### 2.8. Cell Migration

After HBMMSCs reached 80% of confluence, they were starved overnight in an FBS-free medium. The next day they were placed on the top of Transwell inserts with a pore size of 8 µm (Corning, Corning, NY, USA). In the wells 500 µL of the starvation medium was added, supplemented with 50 µL of exosome resuspension and/or fibrin gel components. For the positive control, 20% of FBS was used instead of exosomes. DMEM, without any addition of FBS, was used as a negative control. Inserts with stem cells were immerged into the wells and left in the incubator for 24 h. Cells were fixed in 3.7% formaldehyde, permeabilized by 100% methanol and stained with crystal violet (Acros Organics, Geel, Belgium). The inside of each insert was cleaned using cotton swabs. Cells that migrated from the inside of the insert to the other side of the membrane were visualized using an inverted microscope (CKX53; Olympus, Shinjuku, Tokyo, Japan). Four pictures were taken from each insert, and cells were counted therein using ImageJ.

### 2.9. Cell Proliferation

HBMMSCs were seeded in a 96-well plate at the concentration of 10^3^ cells/mL. After 6 h, the medium was exchanged in a total volume of 100 µL depending on the group:

1. DMEM + exosomes (10 µL)
2. DMEM + 10% FBS
3. DMEM + 10% FBS + exosomes (10 µL)
4. DMEM + 20% FBS

For the negative control, DMEM without supplements was used. Cell proliferation was measured at two different time points: 24 h and 48 h. To detect cells, the tetrazolium salt WST-1 (Roche, Basel, Switzerland) was added. Cells were incubated with WST-1 for 4 h at 37 °C and 5% CO_2_. The reaction produced a color change, which is directly proportional to the amount of mitochondrial dehydrogenase, which is reflective of cell number. The absorbance was measured at 450 nm and 630 nm using Synergy HT microplate reader (Biotek, Winooski, VT, USA).

### 2.10. Statistical Analysis

All experiments were repeated at least three times independently to verify reproducibility. GraphPad Prism version 5.04 was used for the statistical evaluation (GraphPad, La Jolla, CA, USA). The data were expressed as mean ± standard deviation. One-way analysis of variance (ANOVA) was used to determine whether there were any statistically significant differences between groups, and the Student’s t-test was used to compare mean values between individual groups. Bonferroni adjustment was applied for multiple comparisons. The level of statistical significance was set at *p* < 0.05.

## 3. Results

### 3.1. Characterization of Exosomes Derived from Dental Pulp

**Figure 1:**
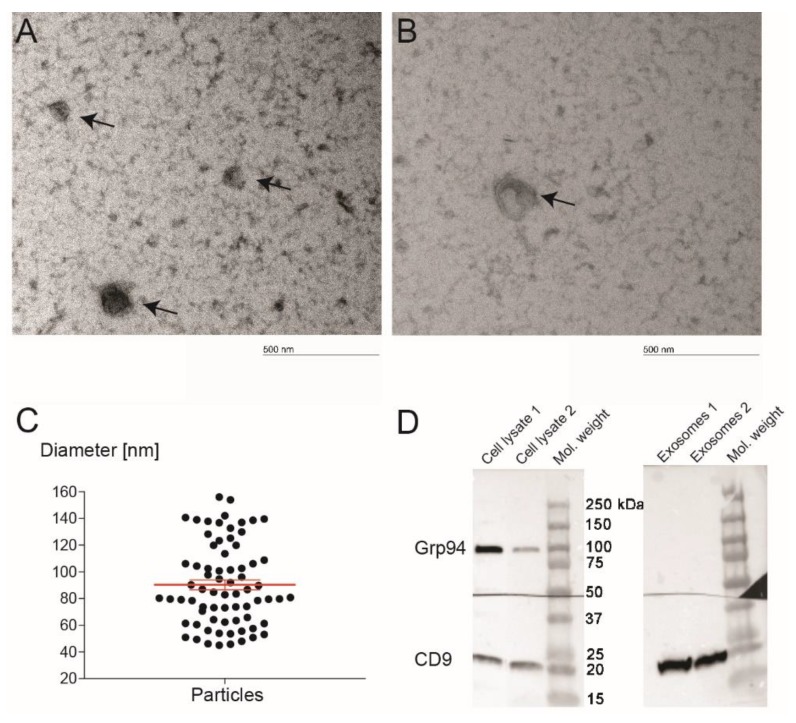
The characterization of exosomes derived from dental pulp. TEM was used to study the structure and the size of exosomes (**A**,**B**). Image of an extracted exosome showing the typical lipid bilayer structure (**B**). The scale bar represents 500 nm. Microscope magnification was 66,000×. It was observed that the diameter of isolated particles was 90 ± 30 nm (**C**). Western blot results showed that the key exosomal membrane protein CD9 was positive for exosomes, while the cell protein Grp94 was negative for exosomes (**D**). The analysis verified that the isolated vesicles were indeed exosomes.

The presence of exosomes was verified by TEM (Figure 1A,B), which showed that the diameter of isolated particles varied from 45 to 156 nm (Figure 1C) with a mean size of 90 nm (95% confidence interval 82 to 97). Proteins from the exosomes and the pulp cell lysate were used for Western blot. Immunoblots presented in Figure 1D show that the samples from exosomes were positive for CD9, the protein that is normally found on the surface of exosomes, and negative for Grp94, an abundant resident endoplasmic reticulum protein that was positive for the cell lysate. 

### 3.2. Uptake of Labeled Exosomes by HBMMSCs 

**Figure 2:**
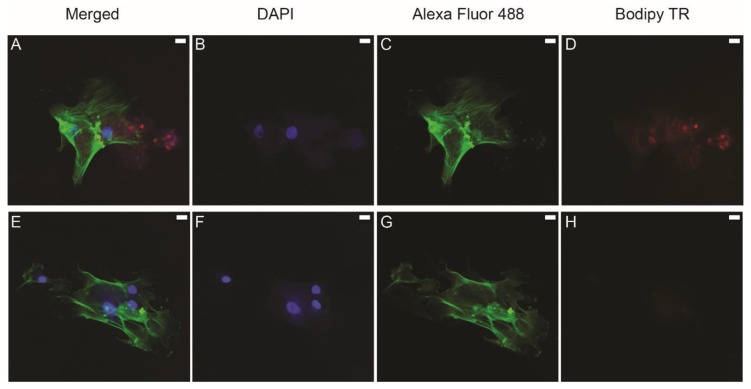
Confocal immunofluorescent analysis of exosome uptake using DAPI to stain the nucleus (**B**,**F**), Alexa Fluor 488 to stain cytoskeletal actin (**C**,**G**), and BODIPY TR to stain exosomes (**D**,**H**). (**A**,**E**) are merged images. Labeled exosomes were isolated from the culture supernatants of dental pulp cells (DPCs). Human bone marrow-derived mesenchymal stem cells (HBMMSCs) were cultured with labeled exosomes. Red dots (**A**,**D**) represent exosomes. The red signal was not expressed in the negative control where non-labeled exosomes were added to the cells (**E**–**H**). Scale bar represents 20 µm.

BODIPY TR ceramide dye allows efficient labeling of the exosomal membrane and was added to the culture medium of DPCs. Labeled exosomes were isolated and added to HBMMSCs. Immunofluorescence image (Figure 2A) shows labeled exosomes (red color) next to the HBMMSCs that have labeled nuclei (blue color) and cytoskeleton (green color). The negative control, the group where non-labeled exosomes were added, did not show exosomes (Figure 2E–H). The images were taken at 40× magnification.

### 3.3. Cell Migration 

**Figure 3:**
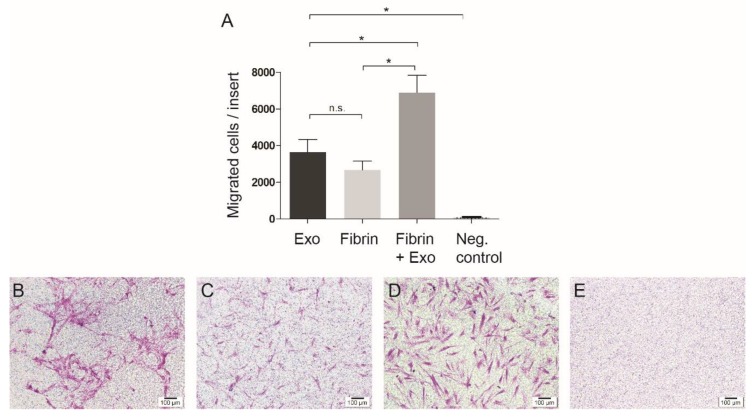
Cell migration was assessed 24 h after the exosomes harvested from human pulp were added to mesenchymal stem cells (MSCs). Quantitative results are presented (**A**). Exosomes (**B**) attracted significantly more stem cells compared to the negative control (**E**). This positive effect of exosomes was enhanced when they were combined with the fibrin gel (**D**) in comparison to fibrin gel alone (**C**). Scale bar represents 100 µm. * *p* < 0.05.

The modified Boyden chamber assay (Figure 3A–E) shows that exosomes induced migration of stem cells (Figure 3B) significantly more than in the negative control (*p* < 0.05). The fibrin alone also triggered the stem cells to migrate (Figure 3C), but the combination of exosomes and fibrin (Figure 3D) showed the highest induction of cell migration (*p* < 0.05). The group with only FBS-free medium did not attract stem cells (Figure 3E). 

**Figure 4:**
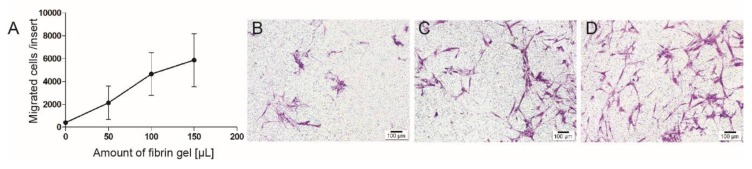
Different amounts of fibrin gel were added to the same amount of stem cells and the migration towards the fibrin gel was measured. Quantitative results are presented (**A**). It was found that 50 µL of fibrin gel (**B**) attracted less cells than the double (**C**) or triple amount of this gel (**D**). The correlation between the amount of fibrin and the migrated cells was almost linear. Scale bar represents 100 µm.

Migration towards fibrin gel is dose-dependent and almost linear between 0 and 150 µL of gel (Figure 4).

**Figure 5:**
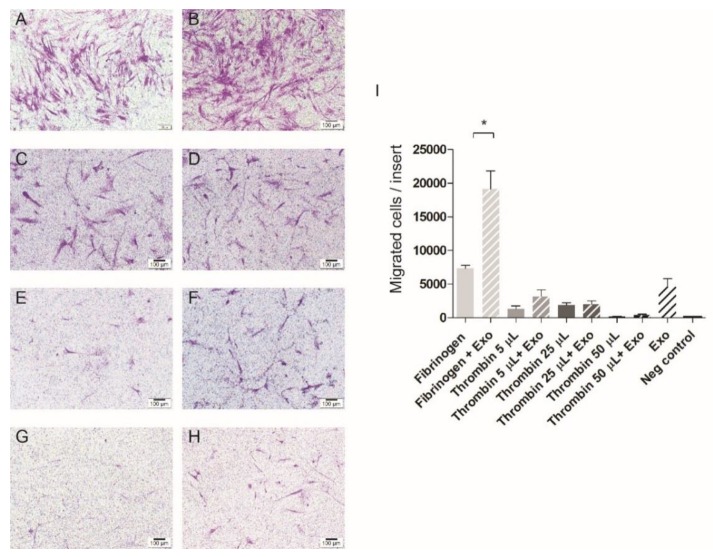
Representative images of the migration towards components of fibrin gel alone (the first column) or supplemented with exosomes (the second column). (**A**) represents a migration toward 50 µL of fibrinogen added to the medium. There was a significant increase (* *p* < 0.05) if the same amount of fibrinogen was combined with 50 µL of exosomes resuspended in phosphate-buffered saline (PBS) (**B**). Thrombin (**C**,**E**,**G**) showed a much lower migration induction potential. Even when exosomes were added, the migration did not change significantly (**D**,**F**,**H**). Scale bar represents 100 µm.

One component of the fibrin gel, fibrinogen, showed high migration potency and the effect was even stronger in combination with exosomes (Figure 5A,B). Thrombin, the other component, attracted cells at low concentrations, but to a much lower extent. The addition of exosomes to thrombin did not surpass the migration potential of exosomes alone (Figure 5C–I).

### 3.4. Cell Proliferation

**Figure 6:**
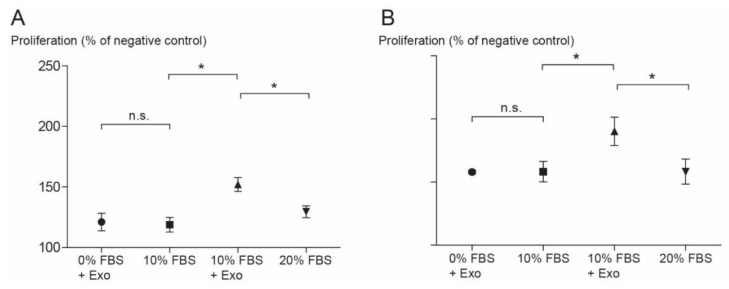
HBMMSCs were stimulated with 10% of exosomes and cell proliferation was measured after 24 h (**A**) and 48 h (**B**). The results are expressed as a percentage of the values from the negative control (only Dulbecco’s modified Eagle’s medium or DMEM). The values in the group that contained only exosomes were similar to the 10% fetal bovine serum (FBS) group; the difference between those two groups was statistically non-significant. The combination of exosomes and 10% FBS enhanced the proliferation more than the addition of 20% FBS did (* *p* < 0.5). The same trends were observed after 24 h and 48 h. Values are presented as the mean ± SD.

To study the effect of exosomes on cell proliferation, HBMMSCs were co-cultured with 10% of exosome resuspension. Cell proliferation was strongly enhanced by adding exosomes, even when the exosomes were without any FBS, both after 24 h and 48 h of stimulation (Figure 6A,B). 

## 4. Discussion

Under the conditions of the current study, exosomes harvested from human dental pulp cells enhanced proliferation and attraction of human bone marrow-derived stem cells. In addition, there was an additive effect of the fibrin gel and the exosomes on cell migration. 

The current data were obtained from in vitro experiments using human bone marrow-derived stem cells and dental pulp cells extracted from human teeth. Consequently, no final clinical conclusions can be drawn. However, our experimental set-up mimics the clinical situation. We used human bone marrow-derived mesenchymal stem cells because in clinics we mostly rely on the recruitment of patients’ endogenous cells from the blood [28]. Moreover, the application of the fibrin product is clinically established and is already authorized in the European Union [29].

Exosomes play a major role in multiple aspects of cell-to-cell interaction [30,31]. Our results demonstrate that exosomes from the human pulp promote cell migration and proliferation of stem cells. This is in line with a previous study on cancer cells that clarified the increase in cell migration by delivery of some ECM molecules through exosomes [32]. In addition, the secretome from MSCs showed a positive effect on angiogenesis and chemotaxis of leukocytes [33]. However, further work is needed to characterize the protein and miRNA profile of the exosomes from the pulp to better understand the underlying mechanisms.

Dental pulp is a unique epithelium-free soft tissue in a hard tissue shell. It is characterized by a variety of cell types including immune cells, odontoblasts, fibroblasts, and dental pulp stem cells [34]. A recent study showed that odontoblasts secrete exosomes protecting cells from apoptosis, which is particularly important in an inflamed state [35]. It is well-known that fibroblasts and endothelial cells have an important role in dentin repair and pulp healing [36,37]. Stem cells, however, represent only between 0.11% and 0.40% of the pulp cells [38]. Hence, the exosomes used in this study represent the exosomes from the entire dental pulp cell population and are not specific for dental pulp stem cells. The advantage of this approach is that no cell sorting or other time-consuming manipulation is needed. Moreover, the pulps from non-infected extracted teeth represent an abundant source for exosomes, which upon isolation can easily be preserved and stored by freezing or lyophilization [39]. Nevertheless, more studies are needed to further understand the effects of the cell source on exosome activity in different clinical or pseudo-clinical situations.

TEM and surface antigen expression results indicate that our isolated particles from human dental pulp were predominantly exosomes as defined. Extracellular vesicles are described as cell-derived vesicles that are enclosed by a lipid bilayer (Figure 1B). Exosomes are highly heterogeneous membranous nanovesicles ranging from 40 nm to 150 nm in diameter depending on their origin, state, and environmental conditions [40]. Some researchers define exosomes as even smaller (30–120 nm [41] or 30–100 nm in diameter [42]). In our samples, the mean size of particles was 90 ± 31 nm. Similar results are reported in other studies [43,44]. Our results differ to a certain extent from the size range mentioned above [40,41,42] because a few larger vesicles were found, which may be due to imaging technique limitation [41]. In this study, we used negative staining TEM, which is probably the most commonly used methodology due to its simplicity and yielding in high contrast images. Lipid membranes, however, are more susceptible to artifacts in negative staining and prone to fusions or fractures [45], which could in part account for the small number of larger vesicles observed under current conditions. 

The transplantation of dental pulp cells or other stem cells into an empty canal shows the formation of pulp-like tissue [9,10]. However, due to the limitations listed above, this approach will not be implemented in clinics within the near future. A major shortcoming of the cell homing approach, on the other hand, is the possible shortage of stem cells able to regenerate the pulp [12]. Since exosomes from human pulp trigger the migration and proliferation of human bone marrow-derived stem cells (as shown in this study), they could become the perfect tool to overcome this recruitment limitation. Moreover, scaffold instability is one of the main reasons for the failure of regenerative treatment [46]. The available evidence seems to suggest that fibrin is a promising candidate for clinical use in regenerative endodontics [24]. Interestingly, the current results reveal that fibrin gel with no other chemotactic stimuli induced the migration of stem cells. This is in line with a published report, albeit from a completely different field [47]. Fibrinogen is converted to fibrin via the activity of thrombin, but both components per se also show some cell-activating activities [48]. The ability of thrombin to attract monocytes depends on its concentration [49]. In our study, we saw enhanced chemotaxis of mesenchymal stem cells by thrombin at low concentrations. At higher concentrations of thrombin, however, even the addition of exosomes failed to further enhance cell migration. In contrast, fibrinogen alone enhanced migration of cells compared to thrombin to a much higher extent, and the effect was further increased by the addition of exosomes (Figure 5). Taking these results together: a fibrin hydrogel appears to be an ideal delivery system for pulp exosomes since their individual activity to induce migration of MSCs is further enhanced substantially. Nevertheless, future studies should also compare different scaffolds in this respect [24]. Moreover, it is not known whether exosomes offer any true advantage over carefully composed cytokine/growth factor additives to the scaffold [13]. Therefore, as mentioned above, identifying the exosome components responsible for the clinically desirable effects observed under current conditions could also help to identify and compose such purely synthetic protein mixtures.

After the cells migrate into the scaffold, they should differentiate to pulp cells. Catelas and co-workers [50], studying the same commercially available fibrin sealant that we used, reported that the differentiation potential of MSCs increases in gels containing high fibrinogen concentrations. The authors suggested that the promotion of the differentiation might be due to higher concentration of growth factors in fibrinogen component of TISSEEL, especially TGF-β1 and b-FGF. Sirieix et al. agreed that the most important biological factor that could influence the efficacy of the fibrin sealant seems to be the fibrinogen concentration [51]. The fibrin gel we used in this study had a much higher concentration of fibrinogen component than thrombin according to the preparation that was already used and showed positive biological properties in pulp regeneration [22]. How the presence of pulp-derived exosomes in the fibrin gel influence the differentiation of the attracted stem cells will be the focus of future studies. 

## 5. Conclusions

Here we show that exosomes secreted from dental pulp cells induce the recruitment and proliferation of human mesenchymal stem cells. As a delivery system for these exosomes, a commercially available and clinically approved diluted fibrin sealant appeared most promising, since in combination with exosomes, it further enhanced the recruitment of stem cells. Exosomes delivered in an injectable fibrin hydrogel of this nature could thus be a powerful tool for cell-free, cell-homing-based regenerative endodontics and a new way to fill dental hard tissues.
